# The Demirjian versus the Willems method for dental age estimation in different populations: A meta-analysis of published studies

**DOI:** 10.1371/journal.pone.0186682

**Published:** 2017-11-08

**Authors:** Temitope Ayodeji Esan, Veerasamy Yengopal, Lynne A. Schepartz

**Affiliations:** 1 Human Variation and Identification Unit, School of Anatomical Sciences, Faculty of Health Sciences, University of the Witwatersrand, Johannesburg, South Africa; 2 Faculty of Dentistry, Obafemi Awolowo University, Ile-Ife, Nigeria; 3 Department of Community Dentistry, School of Oral Health Sciences, Faculty of Health Sciences, University of the Witwatersrand, Johannesburg, South Africa; Medical University of South Carolina, UNITED STATES

## Abstract

**Background:**

The accuracy of radiographic methods for dental age estimation is important for biological growth research and forensic applications. Accuracy of the two most commonly used systems (Demirjian and Willems) has been evaluated with conflicting results. This study investigates the accuracies of these methods for dental age estimation in different populations.

**Methods:**

A search of PubMed, Scopus, Ovid, Database of Open Access Journals and Google Scholar was undertaken. Eligible studies published before December 28, 2016 were reviewed and analyzed. Meta-analysis was performed on 28 published articles using the Demirjian and/or Willems methods to estimate chronological age in 14,109 children (6,581 males, 7,528 females) age 3–18 years in studies using Demirjian’s method and 10,832 children (5,176 males, 5,656 females) age 4–18 years in studies using Willems’ method. The weighted mean difference at 95% confidence interval was used to assess accuracies of the two methods in predicting the chronological age.

**Results:**

The Demirjian method significantly overestimated chronological age (p<0.05) in males age 3–15 and females age 4–16 when studies were pooled by age cohorts and sex. The majority of studies using Willems’ method did not report significant overestimation of ages in either sex. Overall, Demirjian’s method significantly overestimated chronological age compared to the Willems method (p<0.05). The weighted mean difference for the Demirjian method was 0.62 for males and 0.72 for females, while that of the Willems method was 0.26 for males and 0.29 for females.

**Conclusion:**

The Willems method provides more accurate estimation of chronological age in different populations, while Demirjian’s method has a broad application in terms of determining maturity scores. However, accuracy of Demirjian age estimations is confounded by population variation when converting maturity scores to dental ages. For highest accuracy of age estimation, population-specific standards, rather than a universal standard or methods developed on other populations, need to be employed.

## Introduction

Population-based data on human biological growth and development processes are fundamental for assessing the health status of a community. This includes an understanding of the growth pattern for the children as well as the environmental stresses that disrupt or impede their growth. These stresses are often easy to identify, but data on uncompromised development and growth variation in most populations are surprisingly lacking. Instead, researchers typically compare growth in the population of interest to standards formulated for European or US children. The problems associated with using non population-specific standards are complex, and their application can lead to misrepresentations of health status.

The importance of population-specific growth standards extends beyond their utility in biological anthropology and health research. For many populations in rural Africa birth registry and eliciting date of birth is still a challenge. Occlusal tooth wear and anthropological details can be very useful for identification and aging [1,2]. Data on timing of tooth formation, tooth emergence and dental morphometrics are also needed for forensic purposes, especially with the increasing global incidences of mass deaths and disasters [3,4]. Additionally, tables of tooth emergence chronology are useful when birth records are unreliable or lost, where people seek asylum [5], where specific aging is needed to prevent cheating in age-graded sports competitions, or where individuals seek favourable outcomes in civil or criminal cases [6–10]. The age at death is usually the only biological parameter that can be estimated for unidentified juvenile remains with any degree of accuracy [11]. Beyond this, information from dental development may play a major role in determining many clinical decisions, including choices about treatment options and sequence [12]. In the absence of population-specific standards, data from other regions and populations are used as references, often without considering whether they are appropriate for comparison.

Variation in dental development among populations is reported in the literature [13–17]. The reason for the variation among groups is not fully understood, although several explanations involving the interplay of genetic and environmental factors have been proposed [18]. With increasing globalisation there have been observable changes in the demographic features of many populations as well as changes in their physical profiles [19]. Dental parameters are also evolving, and may be related to observable alterations in nutritional status, socioeconomic status, and genetic admixture. With these transformations it is expected that dental growth and development standards of populations will modify with time.

Another source of variation in the timing of dental development is biological sex. Universally, females in any given population are more advanced in tooth formation than their male counterparts [20–23]. Furthermore, other studies [24–26] found that girls are also ahead of boys in permanent tooth emergence in Northern Irish, Finnish and Iranian children respectively, and similar differences are found for most populations.

The effect of malnutrition on dental development remains controversial, with conflicting results from different studies. Malnutrition is thought to have a greater negative impact on skeletal development than on the forming dentition. A recent study by Elamin and Liversidge [27] on severely undernourished children in South Sudan reported no significant impact of nutrition on dental development. However, studies of African Americans and European Americans [28,29] found that children from high socioeconomic backgrounds had earlier tooth emergence, which was attributed to better nutritional status.

### Age estimation

Different methods have been proposed to estimate dental age using permanent tooth formation. Among these is Demirjian's method formulated on a sample of French Canadian children, which involves the assessment of eight specific stages of tooth formation of the seven left mandibular teeth. Biologic weights, which are numerical and derived using the method described in research on skeletal maturity [30], are assigned to each tooth stage. The weights are added together to give a dental maturity score. Separate tables of dental maturity for males and females are used to convert the maturity scores to dental age [20]. The advantage of the Demirjian method is the objective criteria for describing the stages of tooth development. The methodology gained worldwide acceptability and became the most commonly used method for estimation of dental age [20,21]. Studies using the method on other populations documented patterns of comparatively advanced or delayed dental development [18,31–37]. This led several authors to question the cross-populational validity of Demirjian’s method and to argue for population-specific standards for age estimation [18,36,38,39]

Willems and colleagues [14] modified the Demirjian technique by creating new tables from which a maturity score could be directly expressed in years. The step of converting the maturity score to a dental age was omitted, making the new method simpler to use while retaining the advantages of Demirjian's method. There was also a reduction in the overestimation of dental age, which was not statistically different from zero in a Belgian population [14]. This modification was evaluated for several populations and reported to be more accurate than Demirjian’s method [40–45].

No systematic review has compared the accuracy of the Demirjian and Williams methods for dental age estimation versus chronological age in different populations. This review therefore posed the following research question: Does the Demirjian method for dental age estimation provide a more accurate estimate of chronological age when compared to the Willems method in dental age estimation of different populations? The null hypothesis tested was that there was no difference in the accuracy of the two methods for dental age estimation against chronological age.

## Methodology

### Systematic literature search

The literature search was designed to find both published and unpublished studies on the research question. A three-step search strategy was utilized. An initial limited search of MEDLINE and CINAHL was undertaken, followed by an analysis of the text words contained in the title and abstract, and of the index terms used to describe articles. A second search using all identified keywords and index terms was then conducted across all the included databases. Thirdly, the reference lists of all identified reports and articles were searched for additional studies. Studies published in English and only those published from 1973 onward were considered for inclusion. This systematic review is registered with PROSPERO International prospective register of systematic reviews with registration number CRD42016029995. The protocol (under modification) can be accessed via the following website.

http://www.crd.york.ac.uk/PROSPERO/display_record.asp?ID=CRD42016029995

The databases searched included:

MEDLINE, accessed via PubMed: SCOPUS: OVID: Biomed Central: Database of Open Access Journals (DOAJ): Ended: OpenSIGLE and Google Scholar

The search for unpublished studies included:

Hand search: reports: Thesis

Search terms included the following adjusted for the search engine/database used:

- (“Age estimation”) AND (Demirjian OR Willems)
- (“Dental age”) AND (Demirjian OR Willems)
- (“Tooth formation” AND Demirjian)
- Willems AND (“Tooth formation”)

The search was limited up to 28 December 2016.

Studies were eligible for inclusion if they met the following criteria:

- Cross-sectional studies
- Non-cross-sectional studies
- Comparative studies of either method or both methods
- Study focus relevant to the research question
- Full reports (abstracts without full reports not included)
- Study participants ranging in age from 0–18 years

Articles were further excluded according to the following criteria:

- No computable data reported
- For comparative studies, test and control groups not evaluated the same way
- Studies conducted on subjects who were physically or medically compromised and those with developmental anomalies
- Studies conducted exclusively on third molars
- Studies published in any language other than English

Titles and abstracts of identified citations from data sources were scanned by two reviewers (Temitope Esan (TE) and Veerasamy Yengopal (VY)) in duplication, for possible inclusion according to the above criteria. Articles with a suitable title but without a listed abstract were retrieved in full copy. All included articles were judged separately by the authors for possible exclusion with reason or for acceptance, in line with the exclusion/inclusion criteria. Disagreements between authors were solved through discussion and consensus with the third reviewer (Lynne Schepartz (LS)).

### Data collection from accepted trials and analysis

Two reviewers (TE, VY) extracted data from accepted studies independently without being blinded to authors, institutions, journal name or study results. Disagreements between authors concerning data extracted were solved through discussion and consensus. All data were entered in specifically designed data sheets and are reported in the Table of Included Studies (Table 1). The following data were extracted:

1. General important information: First author, year of publication and full article reference, place of trial, age, trial participant characteristics, type of study design
2. Information per test and control group: details of method used, age of participants (dental and chronological age), sex, numbers included

**Table 1:** Table of included studies.

Article	Type of study: Brief details	Details of participants and methods used	Main findings
Amberkova et al. 2014 [50]	Cross-sectional comparative: OPG of 7 left mandibular teeth. Study setting: Macedonia	966 children aged 6–13 analyzed using Willems and Demirjian methods	Willems method most accurate; Demirjian method overestimated chronological age
Asab et al. 2011 [51]	Cross-sectional: OPG of 7 left mandibular teeth. Study setting: Malaysia	905 children aged 6–16 analyzed using Demirjian method	Demirjian method less accurate by overestimating chronological age
Bagherpour et al. 2010 [52]	Cross-sectional. Study setting: Iran	311 boys and girls analyzed using Demirjian method	Demirjian method appropriate only for children 9–13 years
Caneiro et al. 2015 [53]	Cross-sectional retrospective: OPG of 7 left mandibular teeth. Study setting: Portugal	564 children analyzed using Demirjian method	Demirjian method not useful in predicting chronological age. Overestimation of dental age
Cavric et al. 2016 [54]	Cross-sectional retrospective: OPG of 7 left mandibular teeth. Study setting: Botswana	1760 children aged 6–23 analyzed using Demirjian method	Demirjian method not useful in predicting chronological age.
Djukic et al. 2013 [55]	Cross-sectional retrospective: OPG of 7 left mandibular teeth. Study setting: Serbia	686 children aged 4–15 analyzed using Demirjian and Willems methods	Demirjian method overestimated chronological age. Willems method provided better accuracy
El Bakary et al. 2010 [42]	Cross-sectional: OPG of 7 left mandibular teeth. Study setting: India	286 children aged 5–16 analyzed using Willems and Cameriere methods	Willems method predicts better than Cameriere method. Hence could be used in Egyptian population
Erdem et al. 2013 [56]	Cross-sectional retrospective: OPG of 7 left mandibular teeth. Study setting: NW Turkey	425 children aged 7–13 analyzed using Demirjian method	Demirjian method overestimated chronological age and hence not suitable for estimating age
Feijóo et al. 2012 [57]	Cross-sectional retrospective: OPG of 7 left mandibular teeth. Study setting: Spain	1010 children 2–16 analyzed using Demirjian method	Demirjian method overestimated chronological age
Flood et al. 2013 [58]	Cross-sectional retrospective: OPG of 7 left mandibular teeth used. Study setting: Australia	504 children analyzed using the 4 Demirjian methods	All methods not accurate in predicting chronological age.
Galic et al. 2011 [59]	Cross-sectional comparative: Setting: Bosnia-Herzegovina	1089 children analyzed using Cameriere. Haavikko and Willems methods	Willems method overestimated chronological age hence not accurate
Hegde et al. 2016 [60]	Cross-sectional observational: OPG of 7 left mandibular teeth. Study setting: India	1200 children aged 5–15 analyzed using Willems I and Willems 2 methods	Willems 1method predicted age of boys more accurately
Ifesanya et al. 2012 [61]	Cross-sectional retrospective: OPG of 7 left mandibular teeth used. Study setting: Nigeria	124 children aged 4–16 analyzed using Demirjian method	Demirjian method overestimated chronological age
Javadinejad et al. 2013 [62]	Cross-sectional retrospective: OPG of 7 left mandibular teeth. Study setting: Iran	537 children aged 3.9–14 analyzed using Demirjian, Willems, Cameriere and Smith methods	Demirjian and Willems methods overestimated chronological age and hence less accurate
Khoja, Fida and Shaikh 2015 [63]	Cross-sectional retrospective: OPG of 7 left mandibular teeth used. Study setting: Pakistan	403 children analyzed using Demirjian, Willems and Nolla methods	Willems method better predicts chronological age
Kirzioglu and Ceyhan 2012 [64]	Cross-sectional retrospective: OPG of 7 left mandibular teeth. Study setting: Turkey	425 children aged 7–13 analyzed using Demirjian, Nolla and Haavikko methods	All three methods not suitable for Turkish children
Koshy and Tandon 1998 [65]	Cross-sectional retrospective: OPG of 7 left mandibular teeth. Study setting: Southern India	184 children assessed using Demirjian method	Demirjian method overestimated chronological age hence not useful
Kumaresan et al. 2016 [66]	Cross-sectional retrospective: OPG of 7 left mandibular teeth. Study setting: Malaysia	426 children aged 5–15 analyzed using Demirjian, Willems and Nolla methods	Demirjian method least precise, overestimated chronological age
Leurs et al. 2005 [67]	Cross-sectional retrospective: OPG of 7 left mandibular teeth. Study setting: Holland	451 children aged 3–17 analyzed using Demirjian method	Demirjian method overestimated chronological age hence not useful
Mani et al. 2008 [41]	Cross-sectional observational: Study setting: Malaysia	214 boys and 214 girls, selected by simple stratified random sampling. OPGs analyzed using Demirjian and Willems methods	Both overestimated chronological age but Willems had better accuracy
Mohammed et al. 2014 [68]	Cross-sectional comparative: OPG of 7 left mandibular teeth. Study setting: South India	660 children aged 6–13 analyzed using Willems, Demirjian, Nolla and Haavikko methods	All methods are reliable in estimating age
Mohammed et al. 2015 [69]	Cross-sectional comparative: OPG of 7 left mandibular teeth. Study setting: India	332 children aged 6–15.99 analyzed using Demirjian and Willems methods	Willems method is the best predictor of chronological age
Nik-Hussein and Kee Gan 2011 [70]	Cross-sectional study: OPG of 7 left mandibular teeth. Study setting: Malaysia	991 children aged 5–15; Willems and Demirjian methods compared for accuracy	Willems method more applicable for estimating dental age. Demirjian method overestimated chronological age
Patel et al. 2016 [71]	Cross-sectional comparative: OPG of 7 left mandibular teeth. Study setting: India	160 children aged 6–16 analyzed using Demirjian, Willem and Greulich and Pyle methods	Willems method can be accurately used in Southern India
Urzel and Bruzek 2015 [72]	Cross-sectional retrospective: OPG of 7 left mandibular teeth. Study setting: France	743 children aged 4–15 analyzed using Demirjian, Willems I, II and Chaillet methods	Willems I method the most suitable when sex and ethnicity are known
Uys et al. 2014 [73]	Cross-sectional retrospective: OPG of 7 left mandibular teeth. Study setting: South Africa	833 children aged 6–16 analyzed using Demirjian method	Demirjian method overestimated chronological age
Ye et al. 2014 [74]	Cross-sectional retrospective: OPG of 7 left mandibular teeth. Study setting: China	941 children aged 7–14 analyzed using Demirjian and Willems methods	Willems method more applicable for estimating dental age. Demirjian method overestimated chronological age
Zhai et al. 2016 [75]	Cross-sectional retrospective: OPG of 7 left mandibular teeth. Study setting: China	1004 children aged 11–18 analyzed using Demirjian and Willems methods	Demirjian method overestimated chronological age but better accuracy with Demirjian method than with Willems method

OPG = Panoramic Radiographs.

There were three outcome measures assessed:

1. The difference in the dental age versus chronological age for the Demirjian method
2. The difference in the dental age versus chronological age for the Willems method
3. The mean age difference using the Demirjian method versus the Willems method

The above outcomes were compared independently for age and sex in different populations as per the included studies.

Datasets were created to facilitate pooling of similar outcomes into a meta-analysis. A dataset was defined as any extracted set of N, mean and standard deviation (SD) for test and control groups. For comparisons of continuous variables (dental age and chronological age), the mean with the SD was used. If the mean was reported without an SD, then attempts were made to obtain an SD from either the standard error of the mean or the 95% confidence intervals. If the standard error (SE) was reported instead of the SD, then the following formula was used [46]:

$$SD=SE\sqrt{N}$$

When making this transformation, the standard errors were from means calculated from within a group and not standard errors of the difference in means computed between the groups.

If studies reported the 95% confidence intervals (CI, with upper limit *CI*_*u*_ and lower limit *CI*_*l*_), then the following formula was used to calculate the SD:

$$SD=\frac{\sqrt{N}({CI}_{u}-{CI}_{l})}{3.92}$$

The above formula applies to larger sample sizes (>60). If the sample size was small or less than 60 in each group then the denominator (3.92) in the formula above was replaced by 4.128. Again, when making this transformation, the confidence intervals were from means calculated from within a group and not standard errors of the difference in means computed between groups [46].

For each dataset, the Mean Difference (MD) for continuous data with 95% Confidence Intervals (CI) and p-values were computed using a fixed effects model that used the inverse variance for continuous data to include studies directly proportionate to their sample size. Statistical significance was set at p<0.05. For computation of all point estimates, the statistical software program Cochrane RevMan version 5.3 was used.

In order to fulfill the criteria of clinical and methodological homogeneity, which allow for pooling of data for meta-analyses, datasets from the accepted publications did not differ in the following minimum set of characteristics: similar characteristics of children, assessment criteria similar in both groups, data collection and measurements similar in both groups.

### Pooling of datasets

The I^2^ test with 95% CI was used to establish whether any statistical heterogeneity existed between datasets that were assumed to be methodologically homogenous. The thresholds for I^2^ point estimates (in %) and upper confidence values were used in order to interpret the test results [46]: 0–40% = might not be important; 30–60% = may represent moderate heterogeneity; 50–90% = may represent substantial heterogeneity; 75–100% = considerable heterogeneity. Identified (clinically/methodologically/statistically) homogenous datasets were pooled using a fixed effects meta-analysis with the Cochrane RevMan 5.3 software.

### Assessment of methodological quality

Quantitative papers selected for this study were assessed by two independent reviewers for methodological validity prior to inclusion in the review using a revised standardized critical appraisal instrument from the Strengthening the Reporting of Observational Studies in Epidemiology (STROBE) Statement [47]. This is a 40 item checklist used for observational studies (cross-sectional, cohort, case-control). Included studies were assessed according to the checklist and papers that achieved a score of at least 28 out of 40 were regarded as having high methodological quality [48].

### Assessment of publication bias risk

Funnel plots were derived from pooled datasets using the Cochrane RevMan 5.3 software. Symmetrical funnel plots indicate no publication bias and asymmetrical plots are an indication of publication bias.

### Statistical analysis

All statistical analyses were done using the Cochrane RevMan 5.3 software. Analysis was done separately for the two methods under review (Demirjian and Willems) with separate analyses of male and female data. The two methods were compared to determine their accuracy. The weighted mean difference (WMD) was used to assess accuracy of the methods in predicting the chronological age of the children. Heterogeneity and between study variability was assessed using the Tau and I^2^ tests. A significant value of Tau (p<0.05) indicates significant heterogeneity. A value greater than 50% for the I^2^ tests (with values ranging from 0 to 100%) is assumed to be significant. The effect sizes of the Demirjian method for different age groups were compared with those from the Willems method using a Student’s t-test. Statistical significant was inferred at p<0.05.

## Results

### Literature search

**Fig 1:**
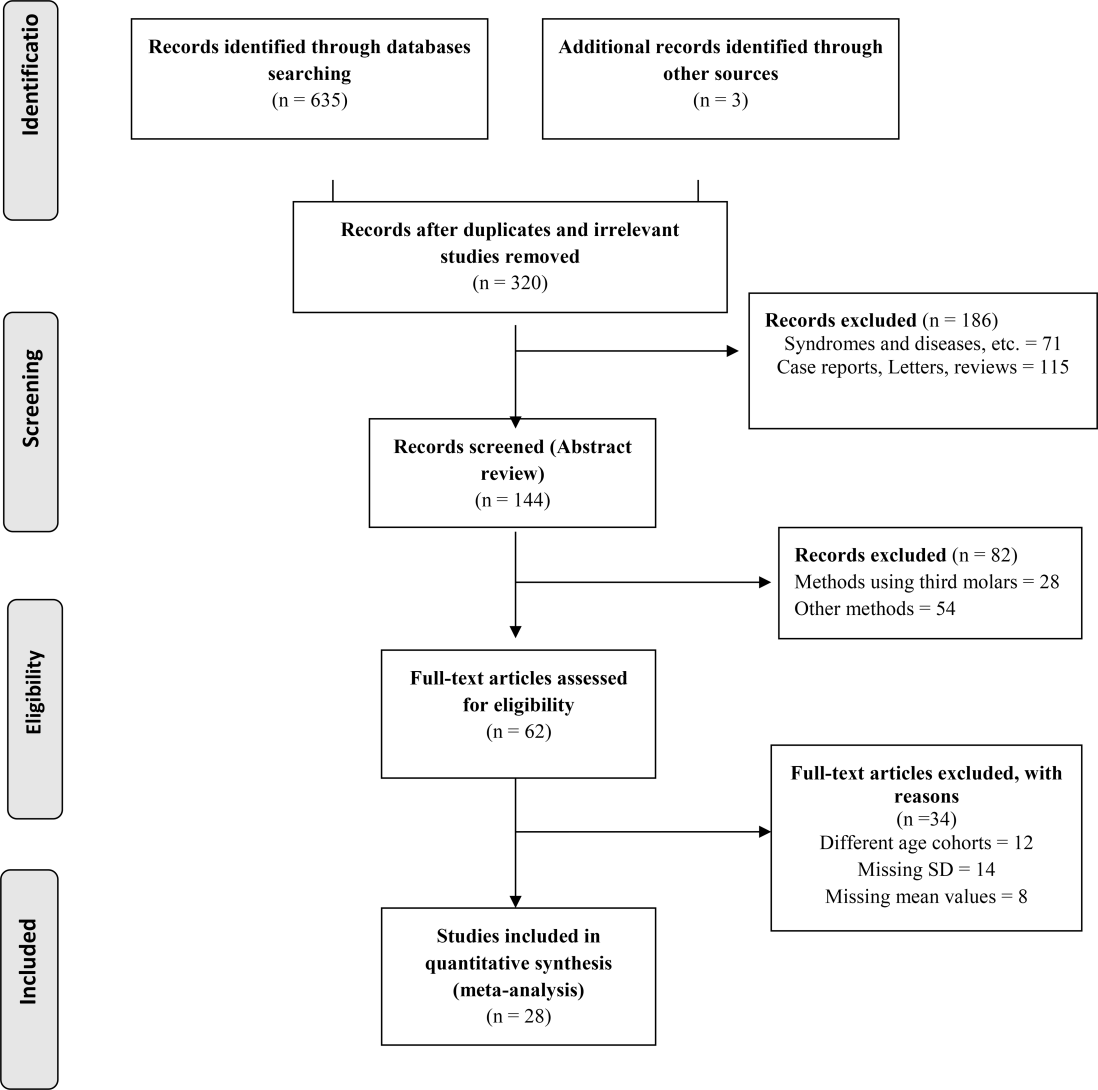
PRISMA 2009 flow diagram for systematic review with meta-analysis.

Fig 1 provides the flow diagram with details of how the identified studies were evaluated for final inclusion in this review. The Preferred Reporting Items for Systematic Reviews and Meta-Analyses (PRISMA) is an evidence-based minimum set of items for reporting in systematic reviews and meta-analyses [49]. PRISMA focuses on the reporting of reviews evaluating randomized trials, but it can also be used as a basis for reporting systematic reviews of other types of research, particularly evaluations of interventions [49]. The common reasons for exclusion were that studies used a different age range (greater than 12 months cohort range, or different age cohort ranges, such as 3.5–4.5), absence of standard deviations, or lack of information regarding the methods for estimating the dental age.

All the cross-sectional studies met the inclusion criteria and were further analysed in this review. Information on these studies is provided in Table 1. Meta-analysis was performed on 28 published articles using the Demirjian and/or Willems methods to estimate chronological age in 14,109 children (6,581 males and 7,528 females) age 3–18 years in studies using the Demirjian method and 10,832 children (5,176 males and 5,656 females) age 4–18 years in studies using the Willems method. Most papers reported that the Demirjian method significantly overestimated the chronological age and was therefore not applicable for use in that specific population. This was observed in studies that used only the Demirjian method and also in studies that compared the Demirjian method to other methods such as the Willems method. The Willems method was found to be a more accurate tool to estimate chronological age (Table 1).

The Strobe 40 item checklist for included cross-sectional studies S1 Table provides the scores obtained when assessing the included studies. The item scores are not intended to be a reflection of the quality of the included papers [76], but are used to provide some insights on the methodological rigor of the individual papers. Most papers achieved scores of around 28, which has been used in previously published studies as an indication of high methodological quality [48].

### Pooled meta-analysis of studies using the Demirjian method to determine difference in the dental age versus chronological age in males and females

**Fig 2:**
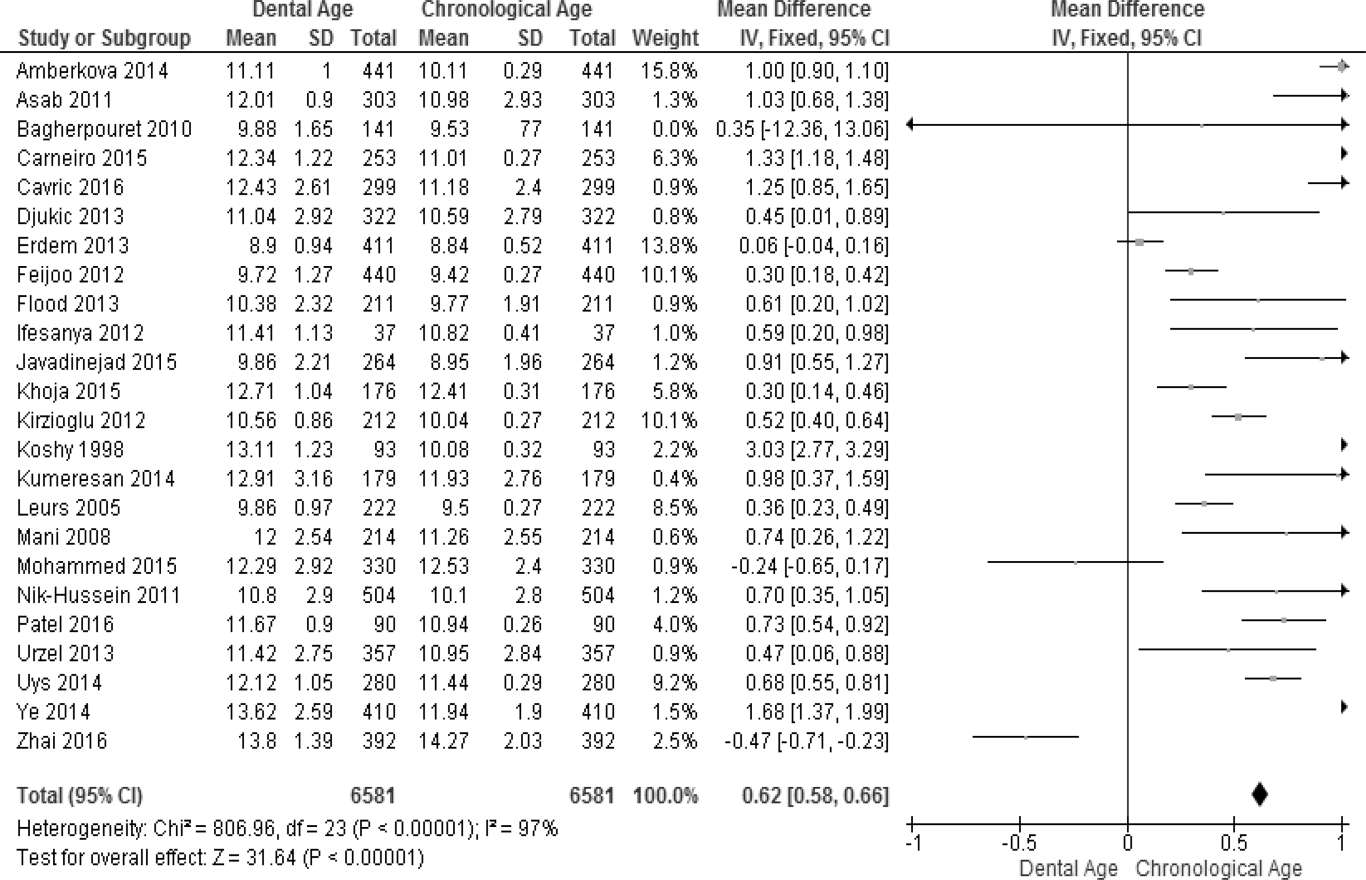
Comparison of dental age and chronological age pooled for males using the Demirjian method.

**Fig 3:**
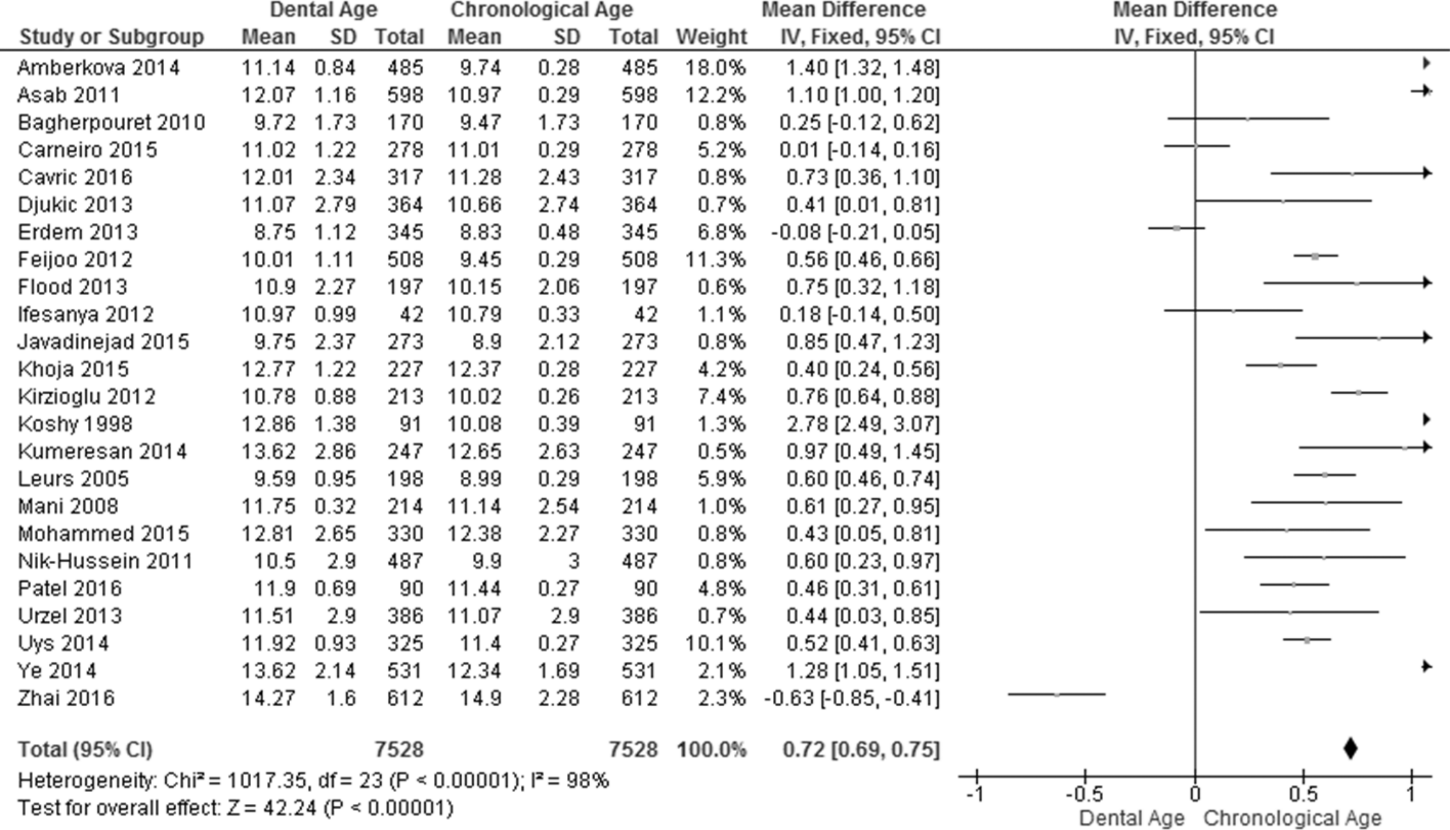
Comparison of dental age and chronological age pooled for females) using the Demirjian method.

The pooled effect estimates for ages 3–18 years in all the included studies were analyzed for males and females and a summary of the results obtained is presented in Figs 2 and 3. Considerable heterogeneity (I^2^ = 97% in males and 98% in females) was found in the pooled analyses for age groups 3–18 years. This can be explained by the pooling together of the ages and studies from different populations that have been found to grow at different rates [77]. Overall, the meta-analysis showed a significant weighted mean difference (WMD) between the dental age and the chronological age in males (WMD = 0.62 years, 95% CI (0.56, 0.66)) and in females (WMD = 0.72 years, 95% CI (0.69, 0.75)). For males (Fig 2), the majority of the studies reported significant overestimation by the Demirjian method. The exception is that of Zhai and colleagues [75], who reported a significant under-estimation of chronological age in males (WMD = -0.63 years, 95% CI (-0.85, -0.41). Three studies [53,56,69] reported no significant difference between dental age estimation and chronological age for males. For females, most studies reported overestimation of the chronological age; only two studies [56,75] reported underestimation of the chronological age (Fig 3).

Meta-analysis of each age cohort of males and females demonstrated that the majority of the age cohorts had considerable heterogeneity (75–100%) with the exception of age cohorts 4 and 16 years in females. The heterogeneity may be due to the pooling of different studies into the meta-analyses. In males, significant overestimation of the chronological age by the Demirjian method was observed in the 3–15 year age cohorts. On the contrary, significant underestimation of the chronological ages was observed in the 16–18 year age cohorts (Table 2). Significant overestimation of the chronological ages of females was observed in all the age cohorts except 3 and 16–18 years where significant underestimation of chronological ages was observed (Table 2).

**Table 2:** Pooled effect estimates (dental age vs. chronological age) for ages 3–18 and sex using the Demirjian method.

Age group	Male					Female				
	Number of studies	n	I^2^ (%)	Effect estimate (95% CI)	SD	Number of studies	n	I^2^ (%)	Effect estimate (95% CI)	SD
3	1	26	NA	**0.57 [0.03, 1.11]**	1.32	1	14	NA	-0.19 [-0.60, 0.22]	0.74
4	4	106	71	**0.61 [0.42, 0.81]**	1.25	4	100	44	**0.28 [0.08, 0.48]**	1.24
5	8	270	93	**1.39 [1.26, 1.51]**	1.28	8	244	82	**1.16 [1.02, 1.30]**	1.36
6	15	614	82	**1.11 [1.04, 1.17]**	1.00	15	608	83	**0.88 [0.81, 0.95]**	1.07
7	19	968	96	**0.76 [0.71, 0.82]**	1.06	19	1084	76	**0.52 [0.46, 0.57]**	1.12
8	20	1360	87	**0.53 [0.46, 0.60]**	1.60	20	1400	76	**0.49 [0.42, 0.55]**	1.51
9	20	1366	82	**0.49 [0.41, 0.58]**	1.95	20	1412	83	**0.57 [0.48, 0.66]**	2.10
10	20	1348	89	**0.75 [0.65, 0.84]**	2.17	20	1367	86	**0.64 [0.55, 0.72]**	1.95
11	21	1556	97	**0.84 [0.77, 0.92]**	1.84	20	1564	91	**0.90 [0.82, 0.97]**	1.84
12	21	1354	95	**0.88 [0.79, 0.96]**	1.94	20	1679	95	**0.87 [0.82, 0.93]**	1.40
13	20	1146	96	**1.08 [1.00, 1.17]**	1.79	19	1420	98	**1.14 [1.08, 1.21]**	1.52
14	17	784	95	**1.06 [0.99,1.14]**	1.30	16	1108	97	**0.60 [0.55, 0.65]**	1.03
15	13	544	95	**0.11 [0.04, 0.18]**	1.01	12	658	96	**-0.20 [-0.27, -0.13]**	1.12
16	4	112	NA	**-1.48 [-1.79, -1.17]**	2.04	5	224	56	**-0.81 [-0.96, -0.66]**	1.39
17	1	76	NA	**-1.95 [-2.17, -1.73]**	1.19	1	148	NA	**-1.52 [-1.67, -1.37]**	1.13
18	1	36	NA	**-2.67 [-2.92, -2.42]**	0.72	1	176	NA	**-2.52 [-2.65, -2.39]**	1.07

Significant values in bold.

### Pooled meta-analysis of studies using the Willems method to determine difference in the dental age versus chronological age in males and females

**Fig 4:**
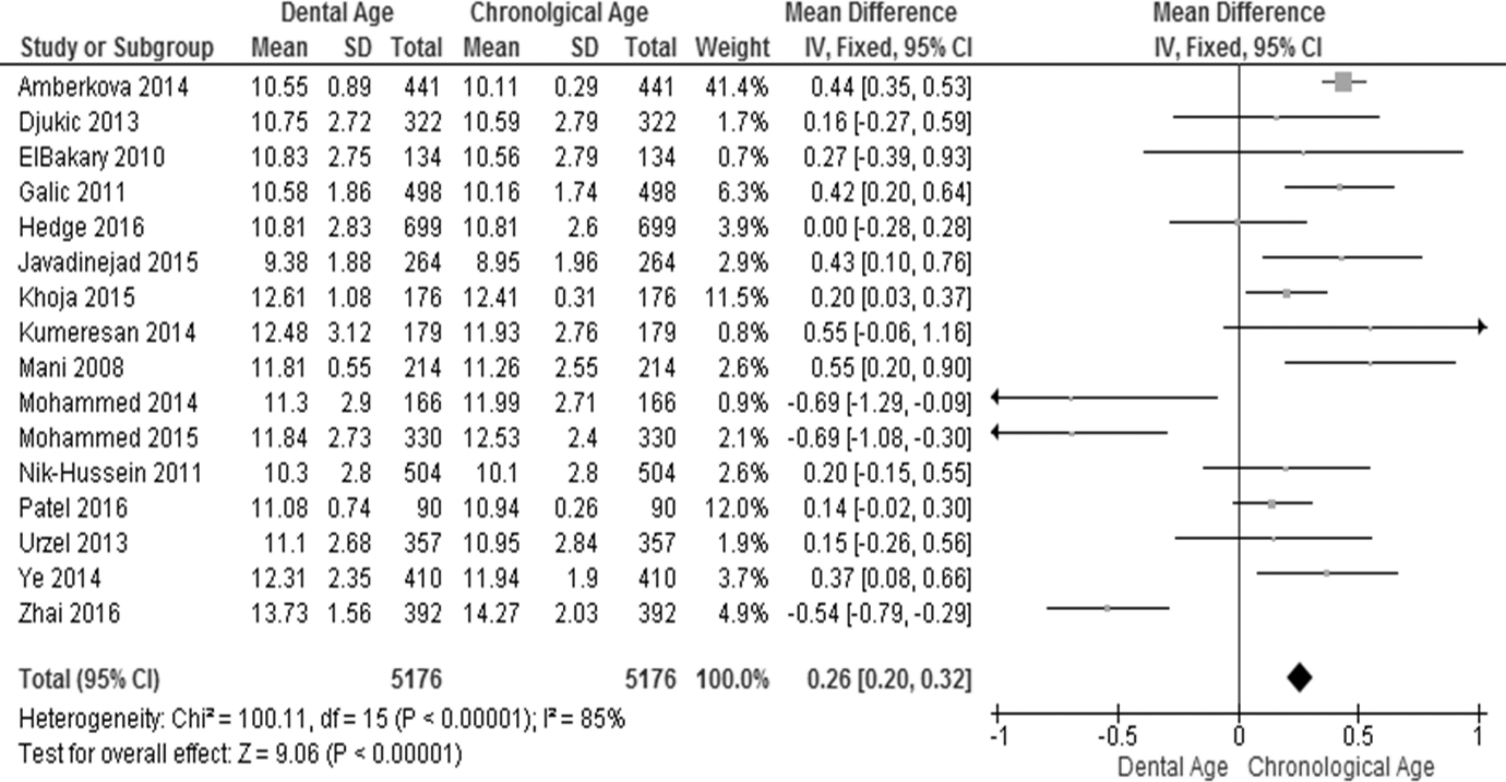
Comparison of dental age and chronological age pooled for males using the Willems method.

**Fig 5:**
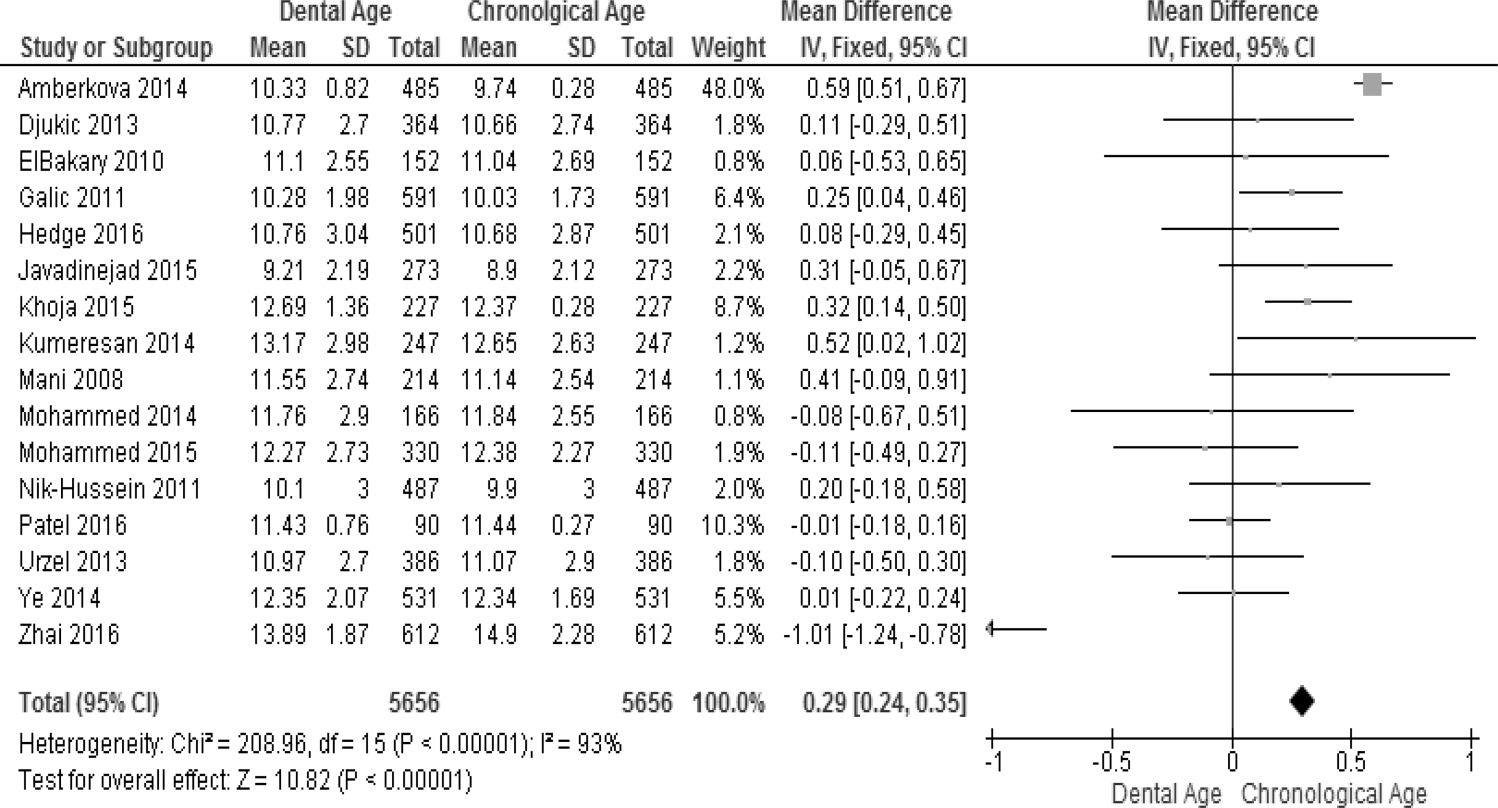
Comparison of dental age and chronological age pooled for females using the Willems method.

The pooled effect estimates of the Willems method for ages 4–18 in all the included studies were analyzed for males and females (Figs 4 and 5). Considerable heterogeneity (I^2^ = 85% in males and 93% in females) was detected in the pooled analyses for age groups 4–18 years. Again this can be explained by the pooling together of the ages and studies from different populations, as mentioned above. The meta-analysis showed significant difference between the dental age and the chronological age in males (WMD = 0.26 years, 95% CI (0.20, 0.32)) and in females (WMD = 0.29, 95% CI (0.24, 0.35)). Six studies reported significant overestimation in males while only four studies reported significant overestimation in females. Furthermore, three studies reported significant underestimation in males, while only one study [75] reported significant underestimation in females. Seven studies of males and 11 of females did not report significant differences (Figs 4 and 5).

Variation in heterogeneity of the included studies was observed for both males and females when the studies were pooled by sex and age cohorts. The I^2^ values ranged from “might not be important” (0–40%) to “considerable heterogeneity” (75–100%) in both males and females. Again, this can be attributed to the pooling together of different ages and populations. Meta-analysis of the age cohorts in males showed significant overestimation in age cohorts 5–14 years, while significant underestimation was found in age cohorts 16–18 years (Table 3). No significant differences were found between the dental ages and chronological ages of children in the age cohorts 4 and 15 years. In females, overestimation of the chronological age was observed in the age cohorts 5–8 and 11–13 years, while significant underestimation was found in the age cohorts 15–18 years (Table 3).

**Table 3:** Pooled effect estimates (dental age vs. chronological age) for ages 4–18 and sex using the Willems method.

Age group	Male					Female				
	Number of studies	n	I^2^ (%)	Effect estimate (95% CI)	SD	Number of studies	n	I^2^ (%)	Effect estimate (95% CI)	SD
4	2	18	0	-0.05 [-0.39, 0.30]	0.91	2	20	0	0.02 [-0.35, 0.40]	0.80
5	4	140	0	**0.31 [0.12, 0.50]**	1.40	4	134	65	**0.45 [0.28, 0.62]**	1.22
6	6	348	73	**0.54 [0.42, 0.65]**	1.33	6	326	83	**0.17 [0.07, 0.26]**	1.07
7	8	510	91	**0.55[0.47, 0.63]**	1.12	8	558	70	**0.18 [0.09, 0.27]**	1.32
8	9	654	89	**0.24 [0.15, 0.33]**	1.43	9	738	55	**0.16 [0.08, 0.25]**	1.43
9	9	764	0	**0.23 [0.15, 0.30]**	1.29	9	694	28	0.07 [-0.03, 0.17]	1.64
10	9	788	53	**0.36 [0.26, 0.46]**	1.74	9	696	55	0.09 [-0.02, 0.19]	1.72
11	10	976	78	**0.30 [0.21, 0.38]**	1.65	10	924	70	**0.19 [0.09, 0.29]**	1.89
12	10	916	97	**0.76 [0.67, 0.85]**	1.69	10	1048	36	**0.13 [0.03, 0.22]**	1.91
13	10	874	97	**0.58 [0.50, 0.65]**	1.38	10	874	99	**0.36 [0.27, 0.45]**	1.65
14	9	574	85	**0.20 [0.08, 0.33]**	1.86	9	764	91	-0.06 [-0.19, 0.06]	2.15
15	8	438	91	0.00 [-0.10, 0.11]	1.36	8	494	79	**-0.21 [-0.33, -0.09]**	1.66
16	2	98	NA	**-1.63 [-2.01, -1.25]**	2.34	3	196	0	**-0.94 [-1.13, -0.74]**	1.70
17	1	76	NA	**-2.15 [-2.46, -1.84]**	1.68	1	148	NA	**-1.64 [-1.77, -1.51]**	0.98
18	1	36	NA	**-2.72 [-3.10, -2.34]**	1.42	1	176	NA	**-2.66 [-2.78, -2.54]**	0.99

Significant values in bold.

### Pooled meta-analysis of studies comparing the Willems and Demirjian methods in males

At age 4 years there was no significant difference (p>0.05) in the effect size between the Willems and the Demirjian methods in age estimation. From age cohorts 5–14 years there were significant differences in the effect estimate between the two methods (p<0.001), with the magnitude of deviation of the dental age from the chronological age significantly greater with the Demirjian method compared to the Willems method (Table 4). It should be noted that the two methods overestimated the chronological ages for these age groups. The Willems method estimated age group 13 accurately, judging from the WMD of 0.00 found in this review. From ages 14–18 years, no significant difference (p>0.05) exists between the effect sizes of Demirjian’s method and the Willems method (Table 4). Overall, the Demirjian method significantly overestimated chronological age compared to the Willems method in males (p = 0.000).

**Table 4:** Comparison of the effect estimates (pooled for age cohorts) of the Demirjian and Willems methods in males.

Demirjian Method				Willems Method				t	p
Age group	n	Effect estimate	SD	Age group	N	Effect estimate	SD		
3	26	0.57	1.32						
4	106	0.61	1.25	4	18	-0.05	0.91	2.14	**0.03**
5	270	1.39	1.28	5	140	0.31	1.40	7.92	**0.00**
6	614	1.11	1.00	6	348	0.54	1.33	7.51	**0.00**
7	968	0.76	1.06	7	510	0.55	1.12	3.55	**0.00**
8	1360	0.53	1.60	8	654	0.24	1.43	3.80	**0.00**
9	1366	0.49	1.95	9	764	0.23	1.29	3.30	**0.00**
10	1348	0.75	2.17	10	788	0.36	1.74	4.30	**0.00**
11	1556	0.84	1.84	11	976	0.30	1.65	7.48	**0.00**
12	1354	0.88	1.94	12	916	0.76	1.69	1.70	0.09
13	1146	1.08	1.79	13	874	0.58	1.38	6.85	**0.00**
14	784	1.06	1.30	14	574	0.20	1.86	9.65	**0.00**
15	544	0.11	1.01	15	438	0.00	1.36	1.45	0.15
16	112	-1.48	2.04	16	98	-1.63	2.34	0.50	0.62
17	76	-1.95	1.19	17	76	-2.15	1.68	0.85	0.40
18	36	-2.67	0.72	18	36	-2.72	1.42	0.19	0.85
**OVERALL**	**6581**	**0.62**	**1.47**	**OVERALL**	**5176**	**0.26**	**1.51**	**13.02**	**0.00**

Significant values in bold.

### Pooled meta-analysis of studies comparing the Willems and Demirjian methods in females

There was no significant difference (p>0.05) in the effect estimate of the Demirjian and the Willems methods at age 4 years. However, significant differences were noted in the effect sizes of the two methods from ages 5–14 years (p<0.001), while no significant differences were noted for ages 15–18 years (p>0.05). Demirjian’s method overestimated chronological age from 4–14 years and thereafter underestimated ages for 15–18 years. The Willems method overestimated dental age from 4–13 years and thereafter underestimated the chronological age from 15–18 years (Table 5). Overall, the Demirjian method significantly overestimated the chronological age of the females compared to the Willems method (p = 0.000).

**Table 5:** Comparison of the effect estimates (pooled for age cohorts) of the Demirjian and Willems methods in females.

Demirjian Method				Willems Method				t	p
Age group	n	Effect estimate	SD	Age group	n	Effect estimate	SD		
3	14	-0.19	0.74						
4	100	0.28	1.24	4	20	0.02	0.80	0.90	0.37
5	244	1.16	1.36	5	134	0.45	1.22	5.03	**0.00**
6	608	0.88	1.07	6	326	0.17	1.07	9.67	**0.00**
7	1084	0.52	1.12	7	558	0.18	1.32	5.48	**0.00**
8	1400	0.49	1.51	8	738	0.16	1.43	4.89	**0.00**
9	1412	0.57	2.10	9	694	0.07	1.64	5.50	**0.00**
10	1367	0.64	1.95	10	696	0.09	1.72	6.30	**0.00**
11	1564	0.90	1.84	11	924	0.19	1.89	9.21	**0.00**
12	1679	0.87	1.40	12	1048	0.13	1.91	11.64	**0.00**
13	1420	1.14	1.52	13	874	0.36	1.65	11.55	**0.00**
14	1108	0.60	1.03	14	764	-0.06	2.15	8.86	**0.00**
15	658	-0.20	1.12	15	494	-0.21	1.66	0.12	0.90
16	224	-0.81	1.39	16	196	-0.94	1.70	0.86	0.39
17	148	-1.52	1.13	17	148	-1.64	0.98	0.98	0.33
18	176	-2.52	1.07	18	176	-2.66	0.99	1.27	0.20
**OVERALL**	**7528**	**0.72**	**1.35**	**OVERALL**	**5656**	**0.29**	**1.48**	**17.36**	**0.00**

Significant values in bold.

### Evaluation of heterogeneity and publication bias

**Fig 6:**
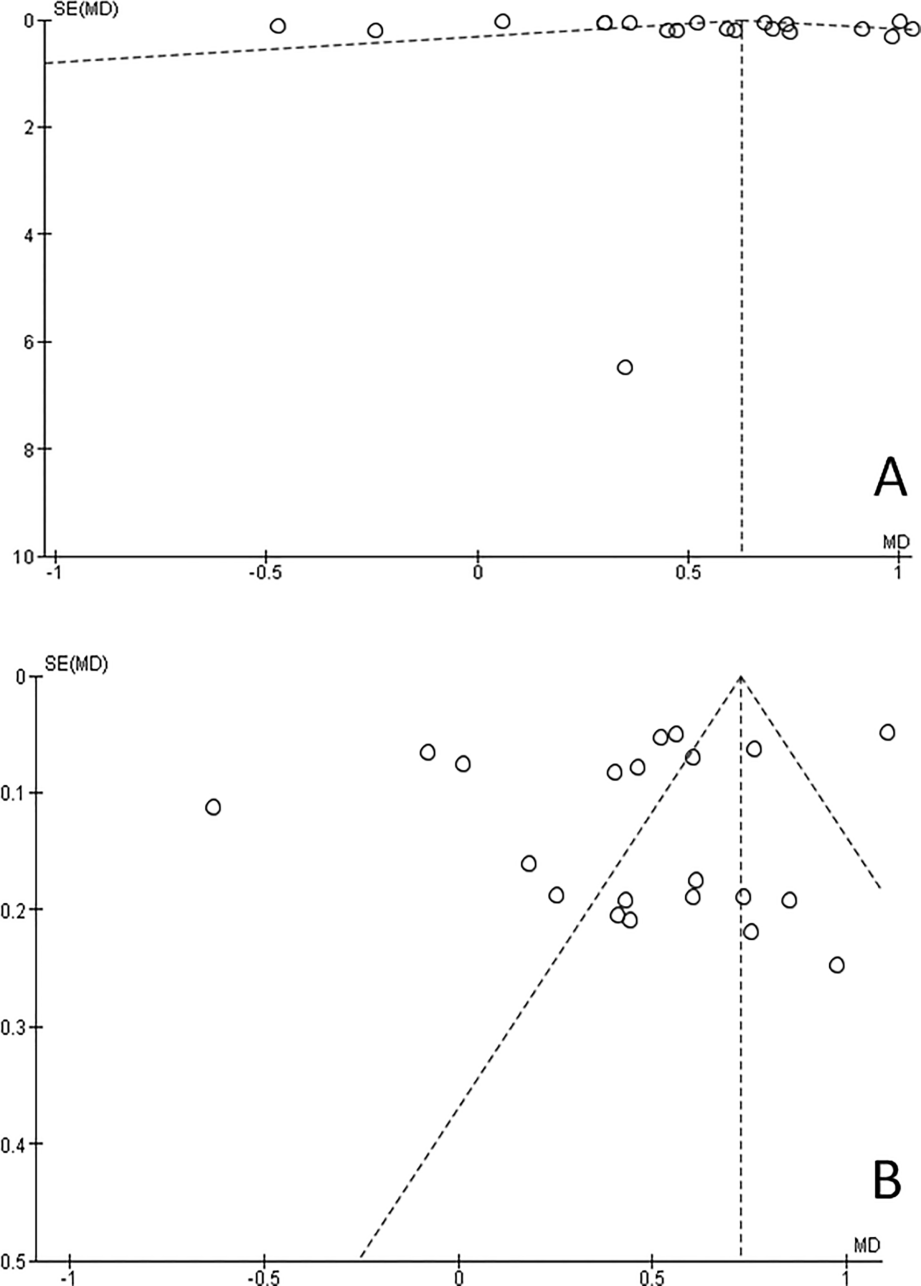
Funnel plots, Demirjian method. Distribution of points across the baseline indicates symmetry. (A) Males. (B) Females.

**Fig 7:**
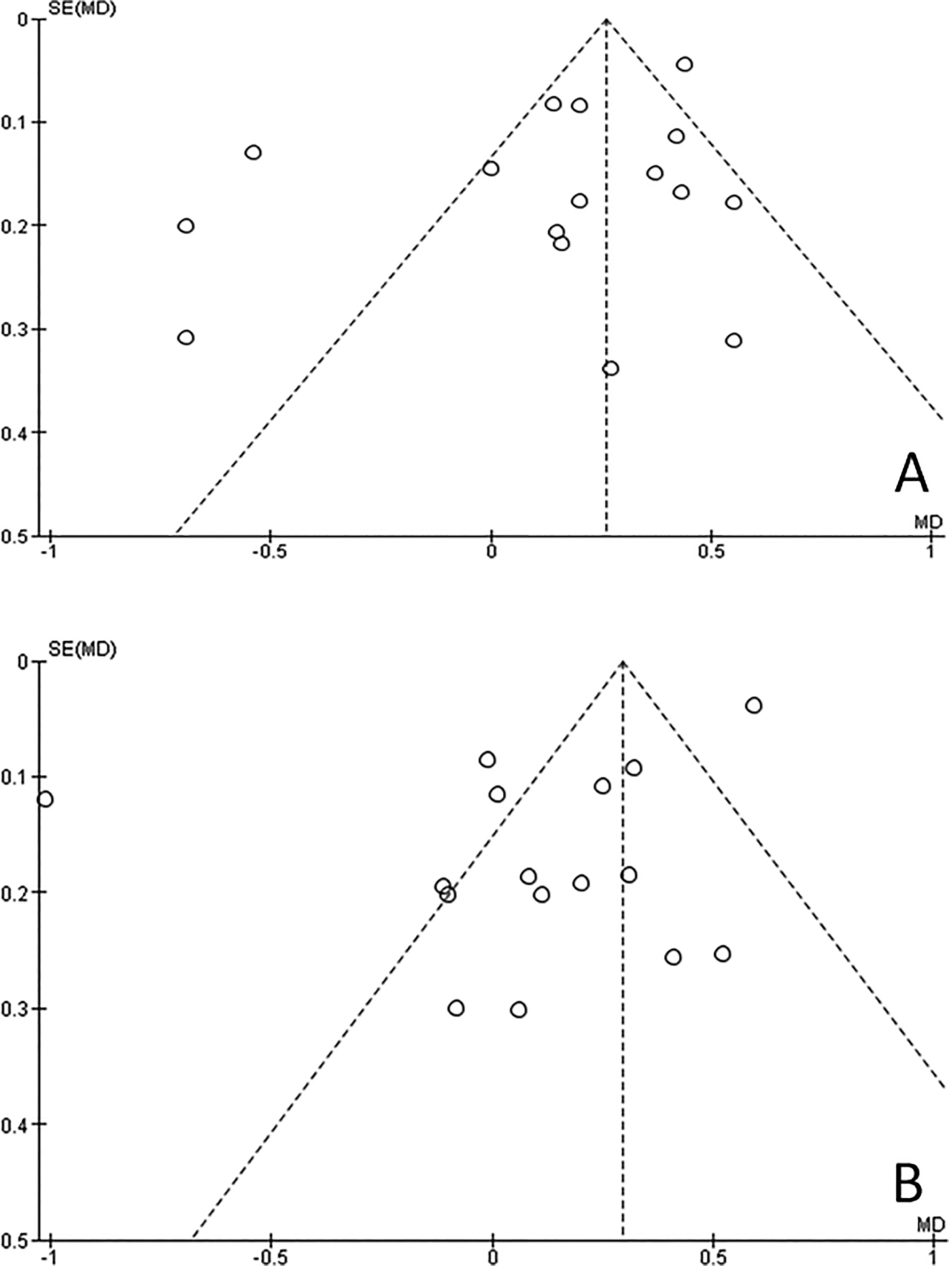
Funnel plots, Willems method. Distribution of points across the baseline indicates symmetry. (A) Males. (B) Females.

No significant difference was noted in the sensitivity test done to determine the influence of individual studies on the overall effect size by omitting each study in turn. Funnel plots were generated to determine the publication bias of the included studies. Visual analysis of the funnel plots does not indicate any evidence of asymmetry as points are distributed across the baseline (Figs 6 and 7).

## Discussion

Standards for growth and development are desirable for forensic, anthropological and clinical purposes [11]. Most methods for assessing growth and development, especially those based on the skeleton, are not highly reliable for estimating age due to the variability stemming from genetic and environmental factors. Dental development is viewed as a more reliable gauge for assessing the age of children and juveniles in forensic and anthropological contexts [20,78], although population variability in dental development has been reported [13–17]. The accuracies of the methods derived from dental maturity, such as the Demirjian and Willems methods, for estimating chronological age across populations are still a subject of debate. Hence this systematic review focused on studies investigating the Demirjian and Willems methods in different populations with the aim of determining the method with a better accuracy.

A limitation of this review is the considerable heterogeneity observed in our results when the results were pooled and also stratified by age and sex. The reason could be due to differences in population characteristic in terms of differences in growth patterns. Furthermore, Demirjian and colleagues stated that their method is based entirely on a French Canadian population and that variation may occur when it is used in other populations. They therefore cautioned that although the stages of the dental maturity scoring system may be universal in application, population differences may affect the accuracy levels when maturity scores are converted to dental ages [20]. This observation highlights the need for population-specific standards for age estimation, especially for forensic and anthropological applications where there are demands for high levels of accuracy.

### Comparison between chronological age and dental age using Demirjian’s method

This review found the Demirjian method significantly overestimates the ages of males and females aged up to 16 years by 0.62 and 0.74 years respectively. This level of overestimation from the Demirjian method makes it unsuitable for forensic purposes in other populations. Other systematic reviews found similar results of age overestimation with Demirjian’s method [48,79]. The overestimation was greater in females than in males. The reason for this difference is not clear from the meta-analysis, but it may be due to varying levels of sexual dimorphism or sex-based differences in environmental stresses.

The underestimation of the chronological age by the Demirjian method in age cohorts 16–18 years in both males and females is due to the non-availability of values for ages 16 years and above in the Demirjian conversion tables of maturity scores to dental age. By that age, all individuals have attained full maturity of the seven tooth (I1-M2) dental sequence. Hence, all ages above 16 years are underestimated.

### Comparison between chronological age and dental age using Willems’ method

This review found no significant mean difference between dental age estimated by the Willems method and chronological age for the total sample. Overall the Willems method overestimated the chronological age of males by only 0.26 years, while it overestimated females by 0.29 years. This pattern is similar to the result for Demirjian’s method where the ages of females were overestimated more than the males. Similar to the Demirjian method, the Willems method cannot be used to estimate chronological age above 16 years because the upper limit of the total maturity score, which is the dental age of 15.77 years, has been achieved. Therefore anyone above the age of 16 years of age is underestimated.

### Comparison between the Willems and Demirjian methods

This is the first systematic review and meta-analysis comparing the Willems and Demirjian methods. Significant differences between dental ages estimated by the two methods were found. The wide gap between the estimates of the two methods is due to the Demirjian method’s significantly overestimating the dental age in all age groups (except for older children aged 15–18 years, primarily due to the constraints of the method, as discussed above).

Based on our results, the Willems method may be used for age estimation for anthropological or forensic purposes in populations where specific reference values are unknown and the levels of accuracy reported here are deemed acceptable. Nevertheless, it is important to emphasize that both methods significantly overestimated chronological age. Hence, our results illustrate that there is a need for population-specific standards for age estimation when the highest levels of accuracy are required.

### Variation in dental development in human populations: Implications for age estimation

The debate is still ongoing whether tooth development is influenced by factors such as nutrition, climate and chronic or infectious diseases. Studies of fluctuating dental asymmetry, thought to be caused by response to stresses, are inconclusive [80,81]. Although tooth size and basic morphology are generally perceived to be relatively immune to major disruptions compared to other growth indicators, the widespread presence of enamel hypoplasias in human populations attests to some level of disruption affecting dental morphology- one counter example among many. The investigation of differences in the *timing* of dental maturation is challenging. The relationship between malnutrition and tooth formation is difficult to evaluate, with some researchers reporting no effect of malnutrition on tooth formation [27,82,83], while others observed a delay in formation [41,84]. Such studies are based on selected proxies of nutritional status such as height, weight and body mass index (BMI). Well-designed studies on severely malnourished children are lacking and constrained by ethical considerations. Recent research on Southern African Black children documents significant differences in the timing of tooth formation in children of different BMI statuses (Esan and Schepartz, n.d.).

Fewer researchers have considered whether the timing of tooth formation varies significantly among human populations. The consistent pattern of variability in overestimation of ages documented by the published studies considered here suggests that variation in the timing of dental development may be influenced by genetic as well as environmental factors. Tables of tooth formation and age of attainment of specific developmental stages from one region of the world may not apply in a different setting, as is clearly demonstrated by our analysis. The documentation of significant variation in dental maturation among human populations, which is growing with expanded research that includes a broader range of populations, needs to be recognized and accounted for in the same way that skeletal and other aspects of growth variation are considered. When the highest levels of accuracy in age estimation are required, population-specific standards need be developed, rather than working toward a global standard.

In conclusion, the Willems method of dental age estimation provides a better and more accurate estimation of chronological age in different populations than the Demirjian method. The Demirjian scoring system has broad application in terms of determining maturity scores, but the accuracies of Demirjian age estimates are confounded by population variation when converting maturity scores to dental ages. Both of the methods reviewed here, when applied to other populations, do not yield a level of accuracy comparable to estimates from population-specific reference data, which should be employed when the highest accuracy is needed.

## Supporting information

S1 File Modified STROBE quality score system. (DOCX)

S1 Checklist PRISMA 2009 checklist. (PDF)

S1 Table STROBE 40 item checklist scores for included cross-sectional studies. (PDF)

S1 Dataset Esan et al systematic review data 19 December 2016. (XLSX)

S2 Dataset Esan et al systematic review pooled results. (XLSX)
