# Supplementary material for: The Demirjian versus the Willems method for dental age estimation in different populations: A meta-analysis of published studies
Source: PLoS One. 2017 Nov 8;12(11):e0186682. doi: 10.1371/journal.pone.0186682 (PMC5678786; doi:10.1371/journal.pone.0186682)
Supplement: S1 File — (DOCX) [file pone.0186682.s001.docx]

| **Modified STROBE Quality Score System**. | |
| --- | --- |
| Criteria items | Score |
|  | (0 to 40) |
| Title and Abstract | □ 0 □ 1 |
| Title and Abstract: Indicate the study design (case-control or cohort study) in the title or the abstract | □ 0 □ 1 |
| Abstract: Provide an informative and balanced summary of the study | □ 0 □ 1 |
| Introduction: Explain the scientific background and rationale for the investigation | □ 0 □ 1 |
| Introduction: State specific objectives, including any prespecified hypotheses | □ 0 □ 1 |
|  |  |
| Methods | □ 0 □ 1 |
| Study Design: Present key elements of study design | □ 0 □ 1 |
| Setting: Describe the setting, locations, and relevant dates, including periods of recruitment, exposure, follow-up, and data collection | □ 0 □ 1 |
| Participants: Give the eligibility criteria of case | □ 0 □ 1 |
| Participants: Give the sources and methods of case ascertainment and control selection | □ 0 □ 1 |
| Participants: Give matching criteria and the number of controls | □ 0 □ 1 |
| Variables: Clearly define all outcomes, exposures, predictors, potential confounders, effect modifiers | □ 0 □ 1 |
| Data sources/Measurement: Give sources of data and details of methods of assessment | □ 0 □ 1 |
| Data sources/Measurement: Describe comparability of assessment methods | □ 0 □ 1 |
| Bias: Describe any efforts to address potential sources of bias | □ 0 □ 1 |
| Study size: Explain and describe the estimation of the study size | □ 0 □ 1 |
| Quantitative variables: Explain how quantitative variables were handled in the analyses | □ 0 □ 1 |
| Quantitative variables: Give group included criteria in the analyses | □ 0 □ 1 |
| Statistical methods: Describe all statistical methods, including those used to control for confounding | □ 0 □ 1 |
| Statistical methods: Describe any methods used to examine subgroups and interactions | □ 0 □ 1 |
| Statistical methods: Explain how missing data were addressed | □ 0 □ 1 |
| Statistical methods: Explain how matching of cases and controls was addressed | □ 0 □ 1 |
| Statistical methods: Describe any sensitivity analyses | □ 0 □ 1 |
| Hardy-Weinberg equilibrium: HWE was assessed | □ 0 □ 1 |
| Hardy-Weinberg equilibrium: HWE of control group was assessed | □ 0 □ 1 |
|  |  |
| Results |  |
| Participants: Report the numbers of individuals at each stage of the study, such as numbers potentially eligible, examined for eligibility, confirmed eligible, included in the study, completing follow-up and analyzed | □ 0 □ 1 |
| Participants: Give reasons for non-participation at each stage | □ 0 □ 1 |
| Participants: Give a flow diagram | □ 0 □ 1 |
| Descriptive data: Give characteristics of study participants (e.g. demographic, clinical diagnosis, ethnicity, sex ratio, etc.) | □ 0 □ 1 |
| Descriptive data: Indicate the number of participants with missing data | □ 0 □ 1 |
| Outcome data: Report numbers in each exposure category, or summary measures of exposure | □ 0 □ 1 |
| Main results: Give unadjusted estimates and confounder-adjusted estimates and their 95% confidence intervals | □ 0 □ 1 |
| Main results: Make clear which confounders were adjusted for and why they were included | □ 0 □ 1 |
| Main results: If relevant, consider translating estimates of relative risk into absolute risk for a meaningful time period | □ 0 □ 1 |
| Other analyses: Report other analyses such subgroups, interactions, and sensitivity analyses | □ 0 □ 1 |
|  |  |
| Discussion | □ 0 □ 1 |
| Key results: Summarize key results with reference to study objectives | □ 0 □ 1 |
| Main results: Report category boundaries when continuous variables were categorized | □ 0 □ 1 |
| Limitations: Discuss both direction and magnitude of any potential bias | □ 0 □ 1 |
| Interpretation: Give a cautious overall interpretation of results considering objectives, limitations, multiplicity of analyses, results from similar studies, and other relevant evidence | □ 0 □ 1 |
| Generalizability: Discuss the generalizability (external validity) of the study results | □ 0 □ 1 |
|  |  |
| Other | □ 0 □ 1 |
| Funding: Give the source of funding and the role of the funders for the present study | □ 0 □ 1 |
|  |  |
