# Supplementary material for: The Demirjian versus the Willems method for dental age estimation in different populations: A meta-analysis of published studies
Source: PLoS One. 2017 Nov 8;12(11):e0186682. doi: 10.1371/journal.pone.0186682 (PMC5678786; doi:10.1371/journal.pone.0186682)
Supplement: S1 Table — (PDF) [file pone.0186682.s003.pdf]

Amberkova et al. 2014	Cross-sectional comparative	27
Asab et al. 2011	Cross-sectional	27
Bagherpour et al. 2010	Cross-sectional	28
Caneiro et al. 2015	Retrospective	27
Cavric et al. 2016	Retrospective	28
Djukic et al, 2013	Retrospective	27
El Bakary et al. 2010	Cross-sectional	29
Erdem et al. 2013	Retrospective	26
Feijoo et al. 2012	Retrospective	26
Flood et al. 2013	Retrospective	28
Galic et al. 2011	Cross-sectional comparative	26
Hegde et al. 2016	Observational	26
Ifesanya et al. 2012	Retrospective	26
Javadinejad et al. 2013	Retrospective	26
Khoja, Fida and Shaikh 2015	Retrospective	27
Kirzioglu and Ceyhan 2012	Retrospective	26
Koshy and Tandon 1998	Cross-sectional	27
Kumaresan et al. 2016	Cross-sectional	25
Leurs et al. 2005	Retrospective	25
Mani et al. 2008	Cross-sectional	28
Mohammed et al. 2014	Cross-sectional comparative	26
Mohammed et al. 2015	Cross-sectional comparative	25
Nik-Hussein and Kee Gan 2011	Cross-sectional	26
Patel et al. 2016	Cross-sectional comparative	25
Urzel and Bruzek 2015	Retrospective	26
Uys et al. 2014	Retrospective	26
Ye et al. 2014	Retrospective	27
Zhai et al. 2016	Retrospective	27
